# Automated Machine Learning Strategies for Multi-Parameter Optimisation of a Caesium-Based Portable Zero-Field Magnetometer

**DOI:** 10.3390/s23084007

**Published:** 2023-04-15

**Authors:** Rach Dawson, Carolyn O’Dwyer, Edward Irwin, Marcin S. Mrozowski, Dominic Hunter, Stuart Ingleby, Erling Riis, Paul F. Griffin

**Affiliations:** Department of Physics, Scottish Universities Physics Alliance SUPA, University of Strathclyde, Glasgow G4 0NG, UK

**Keywords:** magnetometry, atomic, optimisation, machine learning, SERF, caesium

## Abstract

Machine learning (ML) is an effective tool to interrogate complex systems to find optimal parameters more efficiently than through manual methods. This efficiency is particularly important for systems with complex dynamics between multiple parameters and a subsequent high number of parameter configurations, where an exhaustive optimisation search would be impractical. Here we present a number of automated machine learning strategies utilised for optimisation of a single-beam caesium (Cs) spin exchange relaxation free (SERF) optically pumped magnetometer (OPM). The sensitivity of the OPM ($T/\sqrt{\mathrm{Hz}}$), is optimised through direct measurement of the noise floor, and indirectly through measurement of the on-resonance demodulated gradient (mV/nT) of the zero-field resonance. Both methods provide a viable strategy for the optimisation of sensitivity through effective control of the OPM’s operational parameters. Ultimately, this machine learning approach increased the optimal sensitivity from 500 $\mathrm{fT}/\sqrt{\mathrm{Hz}}$ to $<109\mathrm{fT}/\sqrt{\mathrm{Hz}}$. The flexibility and efficiency of the ML approaches can be utilised to benchmark SERF OPM sensor hardware improvements, such as cell geometry, alkali species and sensor topologies.

## 1. Introduction

OPMs have shown impacts across many fields of magnetic sensing, with the potential perhaps being most transformative in the field of magnetoencephalography (MEG). The flexible placement of sensing volumes and favourable operating temperature provide significant advantages over superconducting quantum interference devices (SQUIDs) in many contexts. The sensitivity of commercial OPMs approaches that of SQUIDs while providing functional [1] and longitudinal [2] studies with an important new tool. SERF magnetometers demonstrate sensitivities that approach the low-femtoTesla regime, making this type of zero-field sensor ideal for MEG, although recent work has also demonstrated finite-field sensors attaining the requisite sensitivity for these measurements in the Earth’s field [3,4]. The majority of reported work in SERF sensors for MEG utilise rubidium as the sensing species. Cs is attractive for MEG as the temperature needed to achieve a comparable vapour pressure is lower than that of other commonly used alkalis, rubidium or potassium. To date, few SERF sensors reported in the literature use Cs [5,6] and only a single sensor is known by the authors that operates in a single-beam configuration [7]. As such, the optimal operation parameters of the sensor are not known a priori.

The optimal signal from the SERF sensor has intrinsic complex dynamics in at least five-dimensions contained within the parameters of cell temperature, laser power, laser detuning, modulation frequency and modulation depth. Some experimental parameter configurations have been well-described in the literature [8,9] and others may be modelled accurately [10]. In general, sensitivity is improved by elevating the temperature of the cell to the increase atomic density and subsequently increase spin exchange (SE) collisions. A threshold exists at which the opacity of the cell reduces the transmission of the light through the cell and hence the signal amplitude. Increasing laser power raises the optical pumping efficiency, at the cost of higher intensity noise and broadening of the magnetic resonance (and subsequent reduction of sensitivity). In order to ascertain the best operational parameters for the sensor described here, we have taken an automated approach to optimising the primary experimental parameters with a view to maximising the sensitivity of this device.

Here we present three automated optimisation techniques that have been used independently to assess the best operation parameters based on experimental performance quantified through a chosen cost function *C*. The techniques include a genetic algorithm, a simplified form of gradient ascent optimisation and an open-source machine-learning package that utilises predictive modelling. We present these automated optimisation techniques in the context of a Cs SERF magnetometer to demonstrate use as a generic routine for finding the optimal operating point for a complex sensor.

Beyond the realms of computer science, automated optimisation and machine learning have been utilised across many disciplines [11,12,13,14,15,16], and have found success in quantum and particle physics [17,18,19]. Machine learning has been adopted for the optimisation of experimental parameters for complex systems [20,21,22], where traditional human-intuition-based experimental control is laborious, inefficient, and may not result in the optimal configuration [23].

The optimisation approach applied here has yielded previously unknown configurations of parameters leading to operation of the magnetometer blue-detuned from the optical absorption peak rather than at peak absorption [24]. It has allowed us to create a robust, flexible and fast test environment for benchmarking cells of various buffer gas pressures and different alkali species, which aids sensor development.

## 2. Materials and Methods

### 2.1. Experimental Set-Up

**Figure 1:**
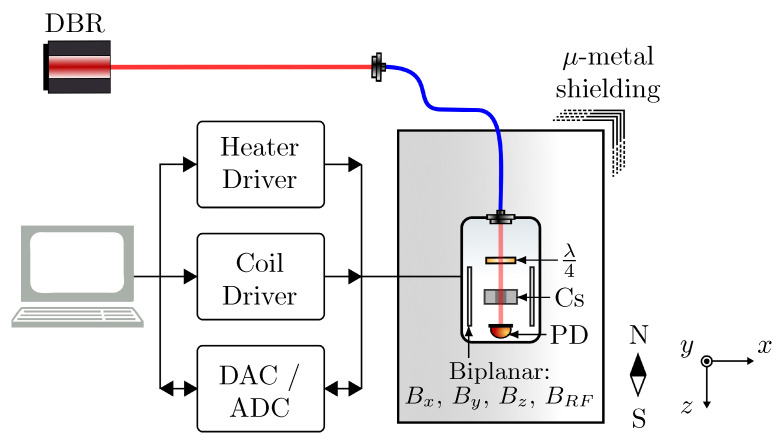
Experimental setup. Elliptically polarised light from a distributed Bragg reflector (DBR) laser close to the $F=4\to F^{\prime }=3$ hyperfine transition of the Cs $D_{1}$ line is fibre-coupled to pass through a micro-fabricated atomic vapour cell [25,26] filled with a saturated vapour of Cs and 211 Torr of nitrogen buffer gas. The cell is heated through resistive heating by square-wave modulated current provided by a custom high efficiency heater driver. Three pairs of biplanar coils, $B_{x}$, $B_{y}$, $B_{z}$, control the static magnetic field along each axis, and an additional modulation coil, $B_{RF}$, allows the application of an oscillating field along the *y*-axis. The static field coils are driven using a custom low-noise current driver [27]. The photodetector (PD) measures light transmitted through the vapour cell. A low nT-level magnetic field environment is provided by a 5-layer μ-metal shield. λ/4, quarter waveplate; Cs, caesium vapour cell; ADC, analog-to-digital converter; DAC, digital-to-analog converter.

The experimental setup is displayed in Figure 1. A distributed Bragg reflector (DBR) laser close to the $F=4\to F^{\prime }=3$ hyperfine transition of the Cs $D_{1}$ line is fibre coupled to the sensor package using a non-magnetic fibre coupler (Schäfter Kirchhoff 60FC-4-M12-10-Ti). Laser power and detuning is controlled by a digital butterfly laser diode controller (Koheron CTL200) through direct control of laser current and TEC temperature. Light polarisation is selected with a miniaturised quarter waveplate (λ/4) that can be manually controlled to allow fine adjustment of polarisation. The beam is incident on a micro-fabricated atomic vapour cell [25], which contains Cs vapour and 211 Torr nitrogen gas. The OPM sensor head [26] consists of all sensing components (cell, optics, PD and coils) in a portable package with external dimensions of 25 mm × 25 mm × 50 mm, which is mounted within a 5-layer μ-metal shield ($10^{5}$ shielding factor) to attenuate the Earth’s magnetic field.

Efforts have been made to reduce the number of magnetic components close to the cell. The cell is mounted on a printed circuit board (PCB), which drives a single 8 Ω non-magnetic aluminium nitride heating resistor. Resistive heating is realised by the application of square-wave current modulation at 274.699 kHz, a frequency far outside the bandwidth of the sensor. The temperature is varied by changing the phase offset of the two square waves that drive a full-bridge class D amplifier. A T-type thermocouple is mounted close to the cell in order to provide temperature feedback.

The cell is mounted at the centre of three biplanar-configuration coil pairs designed using open source coil design package “bfieldtools” [28,29], which control the static magnetic field along each axis. Additionally, a modulation coil along the *y*-axis allows application of an oscillating magnetic field. The static-field coils are driven using a custom low-noise current driver [27]. The light transmitted through the vapour cell is detected using a photodetector with a custom transimpedance amplifier and the signal is digitised via a 16-bit data acquisition system (National Instruments NI USB-6366).

### 2.2. Hanle Resonance

**Figure 2:**
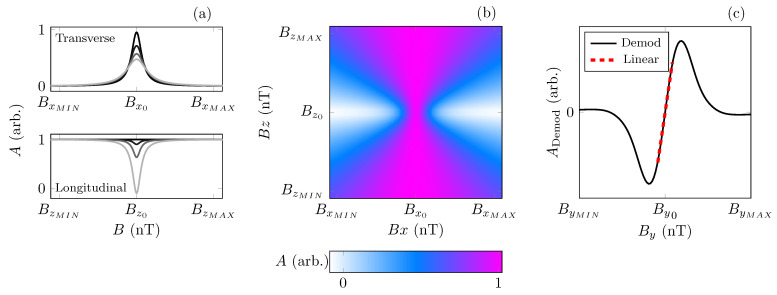
(**a**) (top), Hanle resonances, showing transmission, *A*, across the transverse axis as a function of longitudinal magnetic field; far detuned from zero-field (light line) to zero field, $B_{z_{0}}$ (darkest line). (**a**) (bottom), Hanle resonances, showing transmission, *A*, across the longitudinal axis as a function of the transverse magnetic field; far detuned from zero-field (light line) to zero field, $B_{x_{0}}$ or $B_{y_{0}}$ (darkest line). (**b**), Hanle resonances across two axes. The transverse and longitudinal magnetic fields, $B_{x}\;\&\;B_{z}$, are swept across the *x*- and *z*-axes to generate a 2D landscape of the Hanle resonance. Colour indicates the measured light transmission amplitude (*A*) on the photodetector, normalised with respect to the maximum (1) and minimum (0) transmission. (**c**), modulation of the magnetic field is applied across the *y*-axis as the transverse field, $B_{y}$, is swept from $B_{y_{MIN}}$ to $B_{y_{MAX}}$. The resultant photodiode signal is demodulated and the demodulated amplitude with respect to ($B_{y}$) is shown by the black solid line, the linear sensing region is shown by the red dashed line.

The magnetometer derives its measurement of the magnetic field through the transverse zero-field Hanle resonance [10,30], which manifests as a peak in light transmission through the cell when the atoms experience zero magnetic field, seen in Figure 2a. The static magnetic field on each axis may be swept independently in order to null residual fields [10]. $B_{x}$, $B_{y}$, and $B_{z}$ denote the magnetic field values that are swept along the *x*, *y* and *z* axes, respectively. ${B_{x}}_{0}$, ${B_{y}}_{0}$, and ${B_{z}}_{0}$ denote the magnetic field values that are applied, respectively, to cancel residual static fields and achieve zero-field. The magnetometer is designed to be operated in the SERF regime, which requires elevated temperatures and a low-field environment such that the spin-exchange collision rate sufficiently exceeds the Larmor frequency.

The experimental procedure of the magnetometer is as follows: the magnetic field is swept across the *x*- and *z*-axes to generate a series of longitudinal Hanle resonances with respect to the transverse field, seen in Figure 2b. This two-dimensional “2D” Hanle landscape is fit using Equation (1), which describes the longitudinal Hanle resonance as a function of the field applied in the transverse, in this case *x*, direction [10];

(1) $$S_{2D}=A\left(\frac{\Gamma^{2}+{\left(B_{x}+{B_{x}}_{0}\right)}^{2}}{\Gamma^{2}+{\left(B_{z}+{B_{z}}_{0}\right)}^{2}}\right)-V_{0}\;,$$

where $V_{0}$ is the constant background offset voltage, *A* is the signal amplitude, and Γ is the full-width at half-maximum (FWHM). The point at which the transverse resonance is the sharpest indicates the value of the applied transverse and longitudinal field at which the atoms experience close to zero-field. These fields, ${B_{x}}_{0}$ and ${B_{z}}_{0}$, are applied, effectively zeroing the field in the *x*- and *z*-axes. The final stage steps the field along the *y*-axis to generate a single one-dimensional “1D” transverse Hanle resonance, seen in Figure 2a and [10]. The 1D resonance is fit to the model described as:

(2) $$S_{1D}=A\left(\frac{\Gamma^{2}}{\Gamma^{2}+{\left(B_{y}-{B_{y}}_{0}\right)}^{2}}\right)+V_{0}\;.$$

Subsequently, the magnetic field across the y−axis is swept, with an additional field modulation applied along the same axis at an amplitude ($A_{mod}$) and frequency ($F_{mod}$) determined from Γ. For each value of $B_{y}$, the signal is demodulated. The demodulated line shape, as seen in Figure 2c, shows the linear sensing region (red dashed line), and the gradient (mV/nT) is used to generate the first cost function, Equation (3).

Finally, a free-running measurement of the magnetic field is carried out, allowing the sensor noise floor and hence sensitivity to be characterised. The calculated ${B_{x}}_{0}$, ${B_{y}}_{0}$ and ${B_{z}}_{0}$ fields are applied, effectively zeroing the remaining magnetic field experienced by the sensing atoms across all three axes. Modulation is again applied to the magnetic field along the *y*-axis, and the response of the atoms is measured through the photodetector. Analysis of this measurement through the square root of the power spectral density (PSD) may be scaled by the measured demodulated gradient (mV/nT) to assess the noise floor of the sensor. The power in the noise spectrum across the defined frequency band of interest (5–20 Hz) is calculated, and this serves as the second cost function (Equation (4)) for optimising the OPM.

#### Machine Learning

Machine learning works to identify a global maximum or minimum within a parameter space. Here, we will demonstrate and compare multiple machine learning algorithms (MLAs) that implement supervised learning. Supervised learning refers to providing the MLA with a quantitative measure of performance known as cost [20]. For all techniques, the MLA and experiment are contained within a closed loop where the MLA controls the experiment, which in turn gathers and returns cost information to the MLA. More specifically, the MLA selects the experimental parameters, which are translated to the experiment through control instrumentation. The experiment automatically completes the zero-field resonance measurements in both 2D and 1D, and calculates cost according to the cost function C(ρ). The cost associated with each parameter set is used by the MLA to inform the next set of parameters to sample.

We define two cost functions, $C_{1}\left(\rho \right)$ and $C_{2}\left(\rho \right)$, to optimise in two distinct ways in order to assess which cost function is most effective. $C_{1}\left(\rho \right)$, measured in (mV/nT), is the gradient of the demodulated lineshape as seen in Figure 2c and given by:

(3) $$C_{1}\left(\rho \right)=\frac{\delta A_{\mathrm{Demod}}}{\delta B_{y}}\;,$$

where $\delta A_{\mathrm{Demod}}$ and $\delta B_{y}$ are, respectively, the change in amplitude and magnetic field of the demodulated lineshape within the linear range. $C_{1}$ has been selected as this corresponds to a “sharp” 1D resonance line-shape, that is, a high amplitude with narrow width, which is a good indicator of OPM performance. Thus, $C_{1}$ must be maximised to increase line-shape sharpness and as such a global maximum of $C_{1}$ is desired.

$C_{2}\left(\rho \right)$ is a sensitivity approximation measured directly through analysis of the noise floor. A $\sqrt{PSD}$ is taken to extract a series of frequency dependent amplitude values (X(k)) that are scaled by the demodulated gradient ($C_{1}$) to provide frequency response as a function of magnetic field. The geometric mean of the noise spectrum within our band of interest (5 to 20 Hz) constitutes $C_{2}\left(\rho \right)$, where

(4) $$C_{2}\left(\rho \right)={\left(\prod_{5\leq k\leq 20}^{n}\frac{\delta A_{\mathrm{Demod}}}{\delta B_{y}}X\left(k\right)\right)}^{\frac{1}{n}}\;.$$

By minimising $C_{2}$, which is a measure of the intrinsic noise of the magnetometer in the frequency band of interest, we optimise the magnetic sensitivity. Thus, the location of a global minimum of $C_{2}$ across the parameter space is desired.

Both defined cost functions aim to improve sensitivity, where $C_{2}$ will achieve this directly and $C_{1}$ indirectly.

### 2.3. Optimisation Techniques

For the total number of experimental parameters, *M*, a single set of experimental settings (temperature, laser power, etc.) is defined as $X=(x_{1},\ldots x_{M})$. For each individual set, $X_{i}$, an associated cost $C(X_{i})$ and uncertainty $U(X_{i})$ are found experimentally. All optimisation techniques selected are examples of online optimisation (OO) in which optimisation is implemented concurrently with experimental testing. We employ two evolutionary OO algorithms, a gradient ascent OO and a predictive model-based machine learning algorithm. All optimisation methods continue until 250 sets of parameters are tested, known as the end condition, $N_{end}=250$.

#### 2.3.1. Evolutionary Algorithms

**Figure 3:**
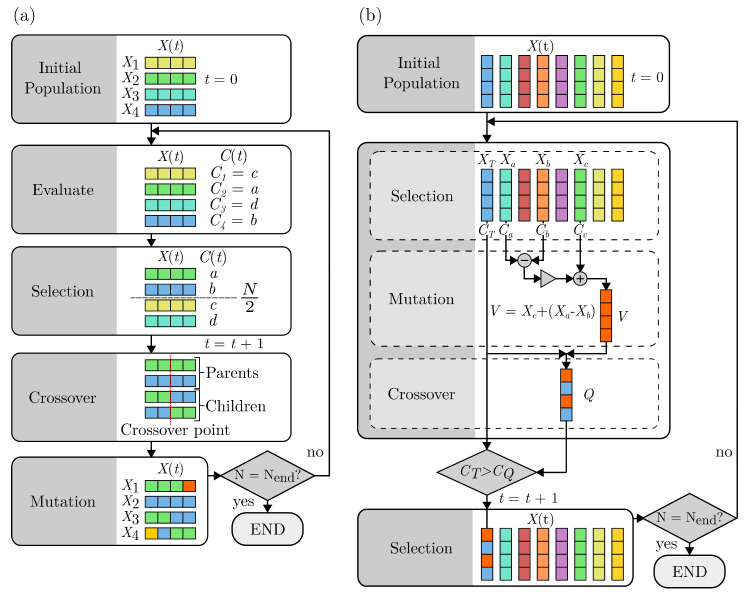
Two evolutionary algorithm processes. (**a**,**b**) share evolutionary elements of initial population formation, selection, crossover and mutation. For both algorithms, the initial population X(t) contains a population of *N* sets of parameter settings. The colour indicates each set of parameter settings. *t*, generation or loop number; t=t+1, the next generation; and C(t), measured cost. Both algorithms repeat until the end condition is met, where the number of sets of parameters tested *N* is equal to 250 ($N_{end}$). (**a**) Genetic algorithm (GA) process. The initial population is generated and evaluated for cost, with individual costs denoted as $C_{i}$. $\frac{N}{2}$ parameter sets are selected for the next generation based on ranked cost. The best performing $\frac{N}{2}$ are used as “parents” to produce “children” sets during crossover with respect to the crossover point. Mutation of individual parameter values randomly occurs in the new population. (**b**) Differential evolution (DE) process. The initial population is generated and evaluated for cost where three random sets $X_{a}$, $X_{b}$ & $X_{c}$ and a target set $X_{T}$ are selected. A new set *V* is created during mutation from the randomly selected sets, and used in a crossover with the target set to make a new set *Q*. $C_{Q}$, the cost of *Q*, is evaluated and measured against $C_{T}$, the cost of the target set. The target set is replaced in a new generation if $C_{Q}>C_{T}$ (for $C_{1}$) or $C_{Q}<C_{T}$ (for $C_{2}$).

Evolutionary algorithms are heuristic search-based approaches to solving problems. The processes of evolutionary algorithms are inspired by nature and biological systems [31], the scheme is shown here in Figure 3. This includes the evaluation of the performance of individuals within a population to inform the selection of a new population mimicking “survival of the fittest”, a crossover of high-performing individuals to imitate reproduction and mutation. Mutation introduces a stochastic component and aims to drive optimisation to a global maximum or minimum. Evolutionary algorithms are commonly used across many types of optimisation problems [32], due to their robust convergence to a solution. However, this convergence time increases with the system complexity. Here, we will implement two evolutionary algorithms, (a) genetic algorithm (GA) and (b) differential evolution (DE) algorithm. The GA process is displayed in Figure 3a. The GA first randomly creates the initial population, X(t), of *N* sets of experimental parameters

(5) $$X\left(t\right)=\left\{X_{1},\ldots X_{N}\right\}\;,$$

where *t* denotes the generation of the population, initially t=0.

All parameters chosen are selected within predefined parameter space limits. Next, we automatically and iteratively evaluate each parameter set, $X_{i}$, through experimental testing and find associated cost C(t) and uncertainty U(t) of the entire population, where, $C\left(t\right)=\left(C_{1},\ldots C_{N}\right)$ and $U\left(t\right)=\left(U_{1},\ldots U_{N}\right)$. The selection of the new generation population, X(t) where t=t+1, is based on the best performing sets of experimental parameters from the previous generation X(t−1). To achieve this, X(t−1) is ranked by C(t−1) with respect to U(t−1) and the best performing $\frac{N}{2}$ sets of parameters are added to X(t). The remaining $\frac{N}{2}$ sets of parameters are created through a crossover. Crossover occurs between sets of parameters from the previous generation to create sets for the new generation, shown in Figure 3a and given by:

(6) $$X{\left(t\right)}_{j}=\left\{x|x\in X_{a}\left(t-1\right)\;\mathrm{if}\;x_{i}\leq CP,x\in X_{b}\left(t-1\right)\;\mathrm{if}\;x_{i}>CP\right\}$$

(7) $$X{\left(t\right)}_{k}=\left\{x|x\in X_{b}\left(t-1\right)\;\mathrm{if}\;x_{i}\leq CP,x\in X_{a}\left(t-1\right)\;\mathrm{if}\;x_{i}>CP\right\}\;,$$

where $X{\left(t\right)}_{j}$ and $X{\left(t\right)}_{k}$ are “children” sets of “parent” $X_{a}\left(t-1\right)$ and $X_{b}\left(t-1\right)$. The crossover point, CP, refers to an individual element, $x_{i}$, of the parent sets. The final step is to introduce random mutation to prevent optimisation for a local minimum or maximum. The new population, X(t), is then evaluated experimentally and the algorithm continues until the end condition is met.

The process of DE deviates from GA as shown in Figure 3b, while maintaining the same evolutionary elements. The initial population of sets of parameters is created as defined in Equation (5) and similarly evaluated to find the associated cost C(t) and uncertainty U(t) of the entire population. The mutation element is incorporated through creation of a new set, *V*, where $V=X_{c}+\left(X_{a}-X_{b}\right)$ and $X_{a}$, $X_{b}$ and $X_{c}$ are randomly selected parameter sets. Crossover occurs between *V* and a randomly selected target set $X_{T}$ to produce an additional set *Q*. *Q* is evaluated experimentally and replaces $X_{T}$ in the new generation where t=t+1, if $C_{Q}$ outperforms $C_{T}$. Lastly, three random sets and a target set are selected from the new population, X(t). The algorithm continues until the end condition is met.

#### 2.3.2. Gradient Ascent

**Figure 4:**
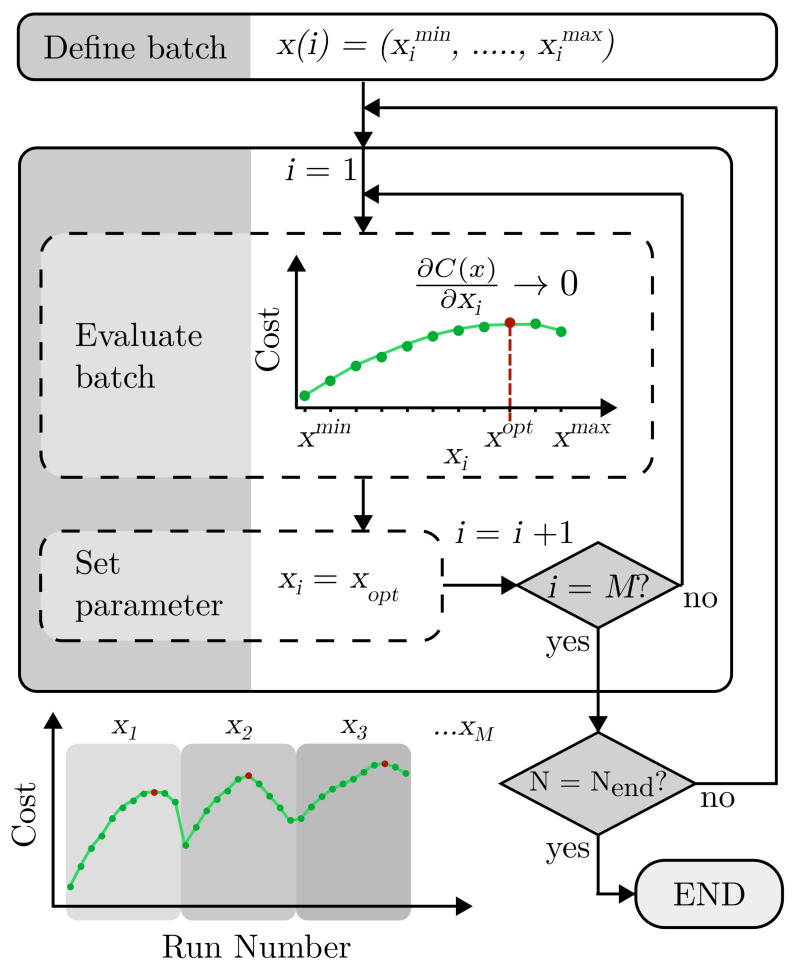
Gradient ascent algorithm process. x(i), a vector value for a single parameter $x_{i}$ ranging from minimum $x_{i}^{min}$ to maximum $x_{i}^{max}$ as defined by parameter space range. *i*, the individual parameter selected. Initially, the first parameter is selected for the first batch i=1. All other parameters are kept constant. The batch is evaluated based on cost, indicated in green, to find where the gradient tends to zero, $\frac{\partial C(\rho )}{\partial x}\to 0$ indicated in red. The corresponding parameter value $x^{opt}$ is then set for this parameter for the next batch, i=i+1. This continues until all parameters are used as batches, for a total number of parameters *M*. The segmented graph shows this process as a function of the run number. This process in turn repeats until the end condition is met, where the number of sets of parameters tested *N* is equal to 250 ($N_{end}$).

Gradient ascent algorithms are a first-order process. As such, the differential of the changing cost C(ρ) is used to inform the learning process [33]. Here, we implement a form of batch gradient algorithm, displayed in Figure 4. Small batches of data are tested to find the optimal parameters based on the gradient of the cost across the batch. Learning occurs between iterations of batches. Batch gradient algorithms guarantee convergence to a local or global maximum or minimum. However, as the batch sizes are pre-defined, some points tested may be redundant, especially compared to stochastic gradient processes with a higher learning rate [34,35,36].

In this context, each batch x(i) is defined as a broad sweep of a single parameter across the full range for that parameter in regular intervals as follows:

(8) $$x\left(i\right)=\left(x_{i}^{min},x_{i}^{min}+n,..x_{i}^{max}\right)\;,$$

where *i* denotes the individual parameter, *n* is the interval for the parameter, and $x_{i}^{max}$ and $x_{i}^{min}$ are the maximum and minimum values of the specific parameter from the defined parameter range. The first batch targets the first parameter only, where i=1. The non-target parameter values are kept constant throughout the batch testing at the previously found optimum, or initially selected randomly. Evaluation of the batch experimentally finds the associated cost for each element of x(i). The cost curve of the batch is used to find where the gradient tends to zero, $\frac{\partial C(\rho )}{\partial x}\to 0$. The value of $x_{i}$ is set to the corresponding parameter value, $x_{opt}$, for the next batch iteration. Each iteration changes the target parameter used for the batch, where i=i+1 after each batch, up to the total number of *M* parameters. One full process of the gradient algorithm occurs after all parameters have been selected as the target parameter, which in turn loops until the end condition is met.

#### 2.3.3. Gaussian Process Regression

The Gaussian process (GP) regression OO method creates a model defining how each experimental parameter relates to the experimentally found cost, known as the cost-landscape. The cost-landscape is formed through training the MLA with data collected by DE for 2*M* sets of parameters. The model generates correlation lengths to indicate how sensitive the cost is to each parameter, where the correlation length is inversely proportional to its influence on cost. The cost-landscape model informs the selection of new parameter values to test. Each iteration informs the model and contributes to defining the noise level of “expected cost” to “found cost”, i.e., the variance of the cost if measured at the same set of parameters many times. For this method, we utilise M-LOOP (Machine Learning Online Optimization Package), an open-source Python-based machine learning toolkit [20], which utilises DE and GP during optimisation. While GP regression is the most sophisticated MLA we employ, Gaussian processes lose efficiency in high dimensional spaces and the computational time required scales with the cube of the number of tests.

### 2.4. Parameters

The parameters, *p*, selected for optimisation are: (1) Cell Temperature T, (2) Laser Power LP and (3) Laser Detuning LD. These parameters are intrinsically linked with complex dynamics as described in Section 4. Each parameter is directly controlled through experimental hardware.

A further two parameters are defined, namely (4) Modulation Amplitude $B_{Mod}$ and (5) Modulation Frequency $\omega_{Mod}$. Both amplitude and frequency of the applied modulated magnetic field influence light absorption and magnetometer performance. These parameters are not directly selected, rather dimensionless factors $A_{Mod}$ and $F_{Mod}$ are defined that are tied to the magnetic resonance line width of the magnetometer response, defined as:

(9) $$\begin{array}{cc} A_{Mod} & =\frac{B_{Mod}}{\Gamma } \end{array}$$

(10) $$\begin{array}{cc} F_{Mod} & =\frac{\omega_{Mod}}{\Gamma \gamma }, \end{array}$$

where total relaxation Γ is equal to the HWHM width extracted from magnetic resonance and γ is the gyromagnetic ratio (3.5×2π Hz/nT for Cs). $B_{Mod}$ and $\omega_{Mod}$ are dependent factors, and the modulation index, $m_{i}$, defines this dependency:

(11) $$m_{i}=\frac{\gamma B_{Mod}}{q\left(P\right)\omega_{Mod}}\;,$$

where q(P) is the nuclear slowing-down factor at high polarisation [37]. It has been shown that the optimal modulation index occurs when $m_{i}=0.5-1$ [38]. All control parameter ranges are defined in Table 1.

## 3. Results

We applied the MLAs presented in Section 2.3 to optimise the sensitivity of a single-beam Cs SERF OPM. Two cost functions ($C_{1}$, $C_{2}$) are utilised to investigate cost function suitability. The number of parameters optimised (M=3: LD, LP, T, M=5: LD, LP, T, $A_{Mod}$, $F_{Mod}$) is varied to demonstrate MLA robustness with respect to optimisation complexity. In total, four independent optimisation schemes are measured:

Scheme 1. Cost = $C_{1}$, M=3
Scheme 2. Cost = $C_{1}$, M=5
Scheme 3. Cost = $C_{2}$, M=3
Scheme 4. Cost = $C_{2}$, M=5

Three MLAs are used per optimisation scheme: (1) Genetic Algorithm (GA), (2) Gradient Descent algorithm (GD) and (3) Gaussian Process Regression (GP). The full parameter space used for all optimisation schemes is defined in Table 1. For equality between optimisation schemes, all methods are initialised with a random set of parameter values, often initially producing no magnetic resonance signal. Each MLA ran until the end condition, requiring 250 sets of experimental settings to be tested, $N_{end}=250$, taking approximately 4 h in total per MLA. Both cost functions were measured during each technique, regardless of the selected cost function, to allow comparison.

To benchmark the optimised sensitivity of all MLAs and optimisation schemes, we first manually optimised through human-intuition-based experimental control. During human optimisation, the operational parameters are manually selected and the subsequent measured sensitivity informs the selection of the next parameters based on intuition. The human optimisation process found an optimal sensitivity of 500 $\mathrm{fT}/\sqrt{\mathrm{Hz}}$, in approximately 4 h.

**Figure 5:**
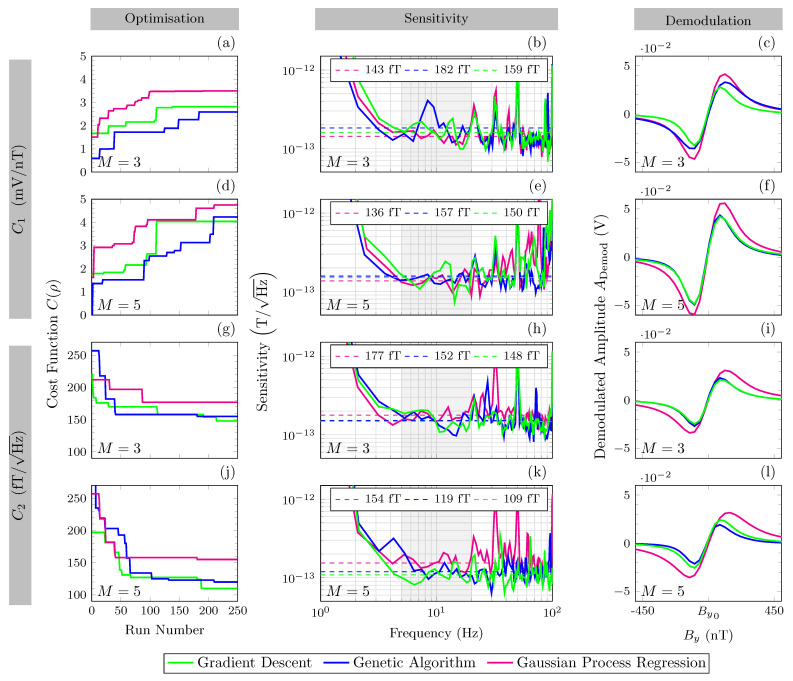
All figure parts contain the following optimisation techniques, gradient descent algorithm in green, genetic algorithm in blue and Gaussian process regression model in pink. *M*, the number of parameters optimised. Row 1 & 3, (**a**–**c**,**g**–**i**), optimisation of 3 parameters (M=3). Row 2 & 4, (**d**–**f**,**j**–**l**) optimisation of 5 parameters (M=5). Row 1 & 2, (**a**–**f**), optimise for maximising cost function $C_{1}$ the demodulated line shape gradient (mV/nT). Row 3 & 4, (**g**–**l**), optimise for minimising cost function $C_{2}$, calculated sensitivity ($T/\sqrt{\mathrm{Hz}}$). Column 1 “Optimisation”, (**a**,**d**,**g**,**j**), show Cost function as a function of run number. The solid line indicates the moving maximum per optimisation technique. Column 2 “Sensitivity”, (**b**,**e**,**h**,**k**), shows corresponding FFT for the optimal parameters found per optimisation technique. Sensitivity is shown as a function frequency (Hz), raw data are shown by solid lines. The frequency band of interest (5 to 20 Hz) is highlighted in grey. Averaged sensitivity in this band is shown by the dashed line (value represented in the key). Column 3, “Demodulation”, (**c**,**f**,**i**,**l**), shows a corresponding demodulated line shape for the optimal parameters found per optimisation technique.

The results of all optimisation schemes for all MLAs are shown in Figure 5. Each row in Figure 5 displays the results for an individual optimisation scheme, with Cost Function C(ρ) and the number of parameters (*M*) indicated accordingly. Progression of each technique can be seen in Figure 5a,d,g,j, where cost is a function of the experimental run number and the moving maximum (for $C_{1}$) or minimum (for $C_{2}$) throughout optimisation run is indicated by the solid line for each MLA. Figure 5b,e,h,k show the corresponding FFT for the optimal parameters found per MLA, with the sensitivity shown as a function of frequency (Hz) in the bandwidth of interest (5 to 20 Hz). Figure 5c,f,i,l depict the corresponding demodulated line shape for the optimal parameters found per MLA.

The optimised cost for each MLA and optimisation scheme with corresponding optimal parameter settings can be seen in Table 2. All optimisation schemes resulted in large cost improvement throughout optimisation. Convergence of optimised values occurred within each optimisation scheme. The mean and standard deviation across all ML techniques within each scheme are summarised below:

Scheme 1. $C_{1}$. All MLAs converged at 2.5±1 mV/nT, equating to a measured sensitivity of 163 $\pm \;20\;\mathrm{fT}/\sqrt{\mathrm{Hz}}$.
Scheme 2. $C_{1}$. All MLAs converged at 4.4±0.4 mV/nT, equating to a measured sensitivity of 147 $\pm \;11\;\mathrm{fT}/\sqrt{\mathrm{Hz}}$.
Scheme 3. $C_{2}$. All MLAs converged at a measured sensitivity of 163 $\pm \;15\;\mathrm{fT}/\sqrt{\mathrm{Hz}}$, equating to a demodulated gradient of 2.2±0.15 mV/nT.
Scheme 4. $C_{2}$. All MLAs converged at a measured sensitivity of 132 $\pm \;23\;\mathrm{fT}/\sqrt{\mathrm{Hz}}$, equating to a demodulated gradient of 2.8±0.9 mV/nT.

The optimum sensitivity of 109 $\mathrm{fT}/\sqrt{\mathrm{Hz}}$ was identified by the gradient descent algorithm (with an uncertainty of $\pm 1\;\mathrm{fT}/\sqrt{\mathrm{Hz}}$ taken from the geometric standard deviation across the frequency band of interest) using five parameters (M=5) and direct optimisation of sensitivity ($C_{2}$). The optimum demodulated gradient of 4.75 mV/nT was identified (with an uncertainty of ±0.03 mV/nT, taken as the linear fitting error across demodulated linear region) by the Gaussian process regression model using five parameters and direct optimisation of demodulated gradient ($C_{1}$).

**Figure 6:**
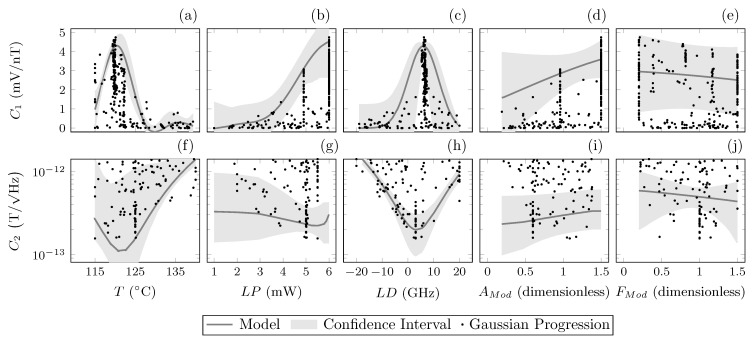
Data and models resulting from the Gaussian process regression model MLA, from a 5 parameter optimisation scheme (M=5). The 5 parameters optimised are cell temperature (T), laser power (LP), laser detuning (LD), modulation amplitude factor ($A_{Mod}$) and modulation frequency factor ($F_{Mod}$). Each part shows a parameter as a function of the cost. Row 1, (**a**–**e**), shows optimisation for cost function $C_{1}$, the demodulated line shape gradient (mV/nT). Row 2, (**f**–**j**) optimisation for cost function $C_{2}$, calculated sensitivity ($T/\sqrt{\mathrm{Hz}}$). Marks indicate measured values from optimisation, solid line indicates the Gaussian process predicted cost-landscape and shaded region indicates the model provided 95% confidence interval of the cost-landscape.

The GP model is the most sophisticated MLA demonstrated in this paper. Due to the nature of the optimisation method, as described in Section 2.3.3, a cost-landscape depicting how each parameter affects the measured cost is produced throughout the optimisation process. Figure 6 shows the measured data for each parameter as a function of cost, for optimisation schemes 2 and 4 (5 parameter optimisations). The parameter cost-landscape model is indicated with a line, and the 95% confidence interval generated by the model is indicated by the shaded region. Many measured points for all parameters lie outside the confidence interval due to the nature of multi-parameter optimisation, where the optimised value of one parameter may produce a poor cost value if other parameters are not optimised. The confidence interval shows the trust region of the models predictive landscape after all measurement has been completed.

## 4. Discussion

The sensitivity of the Cs OPM has been improved by all of the MLAs presented in comparison to human optimisation over comparable run-time. This comprehensive improvement indicates the suitability of automated optimisation methods for experimental parameter optimisation tasks in optically pumped magnetometry.

The use of 3 MLAs allowed for comparison of these techniques to aid recommendations for suitability. In this use case, all techniques appear capable, with no single technique standing out as significantly more favourable. Completing the MLA techniques for a differing number of parameters allows comparison of the robustness of the MLA techniques to the system complexity. Interestingly, the more simple MLAs (GA and GD) proved most successful for direct sensitivity optimisation $C_{2}$, with GD providing the optimal sensitivity value of 109 $\mathrm{fT}/\sqrt{\mathrm{Hz}}$. However, the Gaussian process regression model proved most effective for optimisation of $C_{1}$. This suggests that the Gaussian process regression model was more sensitive to the more stochastic nature of cost function $C_{2}$.

Increasing the complexity, M=5, proved beneficial to both cost functions. As such, the amplitude and frequency of the applied magnetic modulation are tied to magnetometer performance due to their influence on light absorption. Optical noise has a large contribution in this sensor, and this noise decreases with increased absorption. Furthermore, low frequency 1/f noise decreases with increasing modulation frequency. Each five parameter optimisation scheme converged before the end condition, suggesting that, in multi-parameter systems with five parameters, all MLAs are suitable.

The implementation of two cost functions, $C_{1}$ and $C_{2}$, aids identification of the most suitable cost function for this purpose. Table 2 shows relative alignment of the best parameter values between cost functions. The peak sensitivity found indirectly ($C_{1}$) is 30 $\mathrm{fT}/\sqrt{\mathrm{Hz}}$ higher than through direct sensitivity optimisation ($C_{2}$). As such, $C_{1}$ acts as a reasonable proxy for sensitivity optimisation without specifically measuring sensitivity. $C_{1}$ requires less data collection and corresponding sensitivity measurements may be taken after the fact. $C_{2}$ takes longer experimentally and computationally and is more susceptible to extraneous environmental and technical noise. However, 5 parameter optimisation using $C_{1}$ optimised the modulation frequency to a much lower frequency. The subsequent modulation index for these optimised values are also far outside the expected range ($m_{i}>5$). This highlights a key drawback of optimisation using $C_{1}$, that technical noise contributions are not considered.

A benefit of the implementation of the GP is the production of the cost-landscape model that defines how influential each parameter is on performance. From this model, Figure 6, clear trends can be seen that span across both cost functions, for example, the peak in temperature for $C_{1}$ aligns with the trough in $C_{2}$. This is likely due to the increased sensitivity gained when the temperature of the cell has increased atomic vapour density sufficiently to reach the SERF regime. As cell temperature is increased, we see a subsequent improvement of sensitivity up to 120 ${}^{\circ }$C, after which the opacity of the cell is increased by the increasing atomic density, allowing less light to reach the detector.

Figure 6 also shows mirrored trends for laser detuning. However, a deviation between the laser power landscape between cost functions is also present. The peak laser power required for $C_{1}$ continues to increase beyond the defined range, whereas the optimum laser power for $C_{2}$ saturates at 5 mW. This could be due to the increasing laser power detrimentally affecting sensitivity due to intensity noise with increased laser power, which does not degrade $C_{1}$ to the same degree. These trends suggest that either cost function is suitable for optimisation if intensity noise is taken into consideration.

The predicted cost-landscapes for $A_{Mod}$ and $F_{Mod}$ (Figure 6) show broad trends with large confidence intervals, suggesting that the relationship between these parameters and the cost functions are not well-defined. Table 2 shows in the results for optimisation scheme 4 ($C_{2}$, M=5) that the optimised values for modulation amplitude and frequency gave a modulation index within the expected optimal values ($m_{i}=$0.5 − 1). While clear gains in sensitivity were provided by increasing the number of parameters optimised, $C_{2}$ is advised for directly optimising sensitivity while keeping modulation values within expected optimal conditions.

It is interesting to note that the optimal detuning parameter found is positively detuned from the optical absorption peak (Table 2). It appears that the effect of the buffer gas introduces complex optical pumping dynamics in the atomic system, likely tied to depopulation on the F=3 ground state. The results of the MLA techniques show that the detuning and power dependence are non-trivial. These results may vary depending on cell parameters such as the optical path length and buffer gas pressure. The techniques shown here will allow future cells to be characterised in an efficient and comprehensive manner.

With an optimised sensitivity of 109 $\mathrm{fT}/\sqrt{\mathrm{Hz}}$, the ML methods here have aided the tuning of operational parameters of a SERF OPM to facilitate a sensitivity suitable for use in magnetoencephalography.

## Figures and Tables

**Table 1:** Definition of all controlled parameters (*p*) used for optimisation, with corresponding units. Min (*p*), the minimum value for each parameter. Max (*p*), the maximum value for each parameter. Default (*p*), chosen default value if parameter is not directly optimised during optimisation.

Parameter	Min *(p)*	Max *(p)*	Default *(p)*	Unit
Temperature	115	140	-	${}^{\circ }$C
Laser Power	0.5	6	-	mW
Laser Detuning	−20	20	-	GHz
$A_{Mod}$	0.2	1.5	0.5	dimensionless
$F_{Mod}$	0.2	1.5	1	dimensionless

**Table 2:** Optimal parameters found for the following optimisation techniques, Genetic Algorithm (GA), Gradient Descent algorithm (GD) and Gaussian process (GP). The number of parameters tested, *M*, is specified for each optimisation run. T, cell temperature (${}^{\circ }C$). LP, laser power (mW). LD, laser detuning (GHz). $A_{Mod}$, modulation amplitude factor (dimensionless). $F_{Mod}$, modulation frequency factor (dimensionless). $m_{i}$, modulation index (dimensionless). C(ρ) defines the cost function implemented. $C_{1}$ is the demodulated lineshape gradient (mV/nT), with uncertainty taken as the geometric standard deviation across the frequency band of interest. $C_{2}$ is the calculated sensitivity ($\mathrm{fT}/\sqrt{\mathrm{Hz}}$), with uncertainty taken as the linear fitting error across demodulated linear region. Γ is the full-width at half-maximum (FWHM) of the magnetic resonance (nT), with uncertainty taken as the fit error to Equation (2). Values in grey indicate parameters that were not optimised during operation.

MLA	M	C(ρ)	$C_{1}$	$C_{2}$	Γ	T	LD	LP	$A_{\mathrm{Mod}}$	$F_{\mathrm{Mod}}$	$m_{i}$
GD	3	$C_{1}$	2.82 ± 0.03	158.62 ± 1.3	132.51 ± 1.5	119.41	8.24	6.00	0.50	1.00	0.55
GA	3	$C_{1}$	2.59 ± 0.02	182.39 ± 1.4	183.27 ± 2.1	115.00	3.00	5.35	0.50	1.00	0.55
GP	3	$C_{1}$	3.50 ± 0.03	143.40 ± 1.2	168.83 ± 1.6	115.00	8.00	6.00	0.50	1.00	0.55
GD	5	$C_{1}$	4.04 ± 0.02	150.24 ± 1.5	130.06 ± 2.1	118.85	10.77	5.58	1.50	0.30	5.51
GA	5	$C_{1}$	4.23 ± 0.02	157.62 ± 1.3	98.81 ± 2.0	123.00	7.00	5.32	1.48	0.39	4.21
GP	5	$C_{1}$	4.75 ± 0.03	136.30 ± 1.2	147.36 ± 1.2	120.13	6.22	6.00	1.50	0.21	7.82
GD	3	$C_{2}$	2.10 ± 0.02	148.28 ± 1.3	143.36 ± 2.5	117.94	5.88	5.35	0.50	1.00	0.55
GA	3	$C_{2}$	2.35 ± 0.02	152.30 ± 1.3	136.66 ± 1.3	119.00	4.00	4.66	0.50	1.00	0.55
GP	3	$C_{2}$	2.31 ± 0.02	177.40 ± 1.3	192.81 ± 1.6	115.01	3.49	5.57	0.50	1.00	0.55
GD	5	$C_{2}$	2.22 ± 0.03	109.59 ± 1.3	137.70 ± 1.6	118.85	7.69	5.58	0.70	0.80	0.96
GA	5	$C_{2}$	1.95 ± 0.02	119.76 ± 1.2	111.05 ± 2.1	121.00	7.00	5.24	0.97	1.15	0.93
GP	5	$C_{2}$	3.65 ± 0.02	154.81 ± 1.2	203.05 ± 1.6	115.00	3.00	5.50	1.09	1.00	1.20

## Data Availability

All data created during this research are openly available from Pure Data. DOI: https://doi.org/10.15129/e31fe899-cfa6-47a3-8960-fad048c43d23, accessed on 14 February 2023.
